# Chromosome-scale genome assembly of *Camellia sinensis* combined with multi-omics provides insights into its responses to infestation with green leafhoppers

**DOI:** 10.3389/fpls.2022.1004387

**Published:** 2022-09-23

**Authors:** Fen Wang, Baohui Zhang, Di Wen, Rong Liu, Xinzhuan Yao, Zhi Chen, Ren Mu, Huimin Pei, Min Liu, Baoxing Song, Litang Lu

**Affiliations:** ^1^The Department of Life Science and Agriculture, Qiannan Normal College for Nationalities, Duyun, China; ^2^The Key Laboratory of Plant Resources Conservation and Germplasm Innovation in Mountainous Region (Ministry of Education), Guiyang, China; ^3^Horticulture Institute (Guizhou Horticultural Engineering Technology Research Center), Guizhou Academy of Agricultural Sciences, Guiyang, China; ^4^College of Tea Science, Guizhou University, Guiyang, China; ^5^Biomarker Technologies Corporation, Beijing, China; ^6^Peking University Institute of Advanced Agricultural Sciences, Weifang, China

**Keywords:** DuyunMaojian, genome, chromatin interaction mapping, multi-omics, tea green leafhoppers, resistant to insects

## Abstract

The tea plant (*Camellia sinensis*) is an important economic crop, which is becoming increasingly popular worldwide, and is now planted in more than 50 countries. Tea green leafhopper is one of the major pests in tea plantations, which can significantly reduce the yield and quality of tea during the growth of plant. In this study, we report a genome assembly for DuyunMaojian tea plants using a combination of Oxford Nanopore Technology PromethION^™^ with high-throughput chromosome conformation capture technology and used multi-omics to study how the tea plant responds to infestation with tea green leafhoppers. The final genome was 3.08 Gb. A total of 2.97 Gb of the genome was mapped to 15 pseudo-chromosomes, and 2.79 Gb of them could confirm the order and direction. The contig N50, scaffold N50 and GC content were 723.7 kb, 207.72 Mb and 38.54%, respectively. There were 2.67 Gb (86.77%) repetitive sequences, 34,896 protein-coding genes, 104 miRNAs, 261 rRNA, 669 tRNA, and 6,502 pseudogenes. A comparative genomics analysis showed that DuyunMaojian was the most closely related to Shuchazao and Yunkang 10, followed by DASZ and tea-oil tree. The multi-omics results indicated that phenylpropanoid biosynthesis, α-linolenic acid metabolism, flavonoid biosynthesis and 50 differentially expressed genes, particularly peroxidase, played important roles in response to infestation with tea green leafhoppers (*Empoasca vitis* Göthe). This study on the tea tree is highly significant for its role in illustrating the evolution of its genome and discovering how the tea plant responds to infestation with tea green leafhoppers will contribute to a theoretical foundation to breed tea plants resistant to insects that will ultimately result in an increase in the yield and quality of tea.

## Introduction

The tea tree, which has been grown in China for approximately 2,000 years (Kingdom-Ward, 1950), is a very important beverage crop with substantial economic value (Fang et al., 2022). The tea has many functions and effects, such as engendering liquid and allaying thirst, improving eyesight, preventing cancer and other healthy values (Ross and Kasum, 2002; Khan et al., 2008; Tounekti et al., 2013). China and India are the primary producers of tea (Bhattacharyya et al., 2022; Na Nagara et al., 2022; Nyhus Dhillon et al., 2022), and the tea industry in Sri Lanka is the second largest source of foreign exchange (Munasinghe et al., 2017). Tea is becoming increasingly popular worldwide and is now planted in more than 50 countries (Xia et al., 2020b; Yue et al., 2022) because of intriguing flavors (Han et al., 2016; Tan et al., 2019) and health benefits (Zhang et al., 2021b; Ahammed and Li, 2022). DuyunMaojian tea in Guizhou Province is a famous variety that won the Panama World’s Fair Award in 1915 and the cultivation is thought to date back to 1368–1644 AD. The shape of DuyunMaojian tea is like a fishhook and is full of pekoe, which produces high-quality taste. Tea plantations in Guizhou Province occupy 470,000 ha, and ranked first in China by 2022. Duyun Maojian tea plants usually grow in a severe environment at 30° north latitude and an elevation of 800–1,200 m with little sunshine and much cloud and fog. It has a soft aroma, fresh and thick smell, and a sweet aftertaste. Thus, DuyunMaojian tea plant is a good material for breeding. However, tea green leafhoppers (*Empoasca vitis* Göthe) can significantly reduce the yield and quality of tea and cause losses in yield of 11–55% in the absence of effective means of prevention and treatment(Hazarika et al., 2009; Yang et al., 2013). The infestation of tea plants by these leafhoppers can cause physiological and biochemical defense responses, which primarily include the activation of salicylic acid, jasmonic acid, and other signaling pathways(Arimura et al., 2011; Zeng et al., 2019). Thus, we used the DuyunMaojian tea plant genome in combination with transcriptomic, proteomic and metabolomic studies to analyze the differentially expressed genes (DEGs), differentially expressed proteins (DEPs), and differential metabolites that play dominant effects in the response to tea green leafhoppers. We explored the important metabolic pathways in the DEGs, DEPs and differential metabolites that are involved in the molecular mechanism of tea plant responses to infestation with green leafhoppers, which provides a theoretical reference to develop insect-resistant cultivars.

*Camellia sinensis* genomes have been previously published from the cultivars Yukang 10 (Xia et al., 2017), Shuchazao (Wei et al., 2018; Chen et al., 2020; Xia et al., 2020a), Biyun (Zhang et al., 2020a), DASZ (Zhang et al., 2020b), Huangdan (Wang et al., 2021), Longjing 43 (Wang et al., 2020), and Tieguanyin (Zhang et al., 2021a). Two of the genome versions (Xia et al., 2017; Wei et al., 2018) are short-read–based assemblies that are highly fragmented, and the chromosome-scale genome assemblies of Shuchazao, DASZ, Longjing 43 were obtained using PacBio SMRT (Xia et al., 2020a; Wang et al., 2020) and high-throughput chromosome conformation capture (Hi-C) technology. Huangdan and Tieguanyin were assembled using PacBio HiFi (Wang et al., 2021; Zhang et al., 2021a) and Hi-C technology. In this study, we used Oxford Nanopore Technology (ONT) PromethION^™^ (Nicholls et al., 2019) combined with Hi-C technology (Feng et al., 2022; Lin et al., 2022) to assemble the genome of the elite tea cultivar DuyunMaojian. ONT can generate multi-kilobase-long RNA reads and has no bias for GC content and length. Thus, this study will present a theoretical reference for programs to breed tea tree.

## Materials and methods

### Sample collection, DNA preparation and library construction

**Figure 1:**
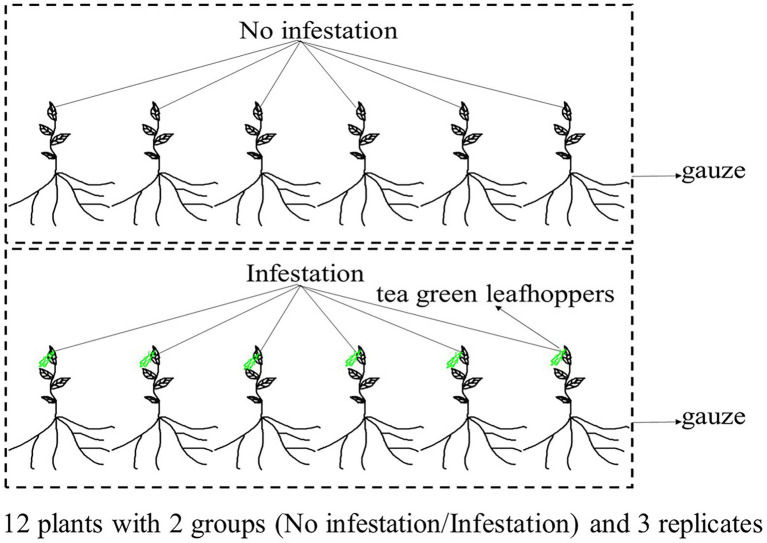
Experimental design. One group of the tea plants were not infested by tea green leafhoppers, and the other were infested by tea green leafhoppers.

An individual plant of DuyunMaojian tea was collected in December 2020 from Duyun City, Guizhou Province, China (N26°20′19″ E107°31′11″). The altitude is 786.1 m. Fresh and healthy leaves were frozen in liquid nitrogen after collection, and stored at –80°C before DNA extraction. DNA was extracted from the leaves using the CTAB method, which were examined by NanoDrop spectrophotometry (Thermo Fisher Scientific, Waltham, MA, United States), Qubit fluorimeter (Invitrogen, Carlsbad, CA, United States) and electrophoresis on a 0.35% agarose gel, and large segments were filtered using the BluePippin^™^ System (Sage Science, Inc., Beverly, MA, United States). We then prepared a library using the large segments of DNA, an ONT Template prep kit (SQK-LSK109; Oxford Nanopore Technologies, Oxford, United Kingdom) and an NEB Next FFPE DNA Repair Mix kit (New England Biolabs, Ipswich, MA, United States). The leaves for transcriptomic, proteomic, and metabolomics analyses were collected from plants same with the genome. Those trees were raised from cuttings planted in Duyun City, Guizhou Province, China (N26°20′19″ E107°31′11″) in November 2018. The leaves were collected from 12 individual trees with similar heights in June 2021. We made sure that 12 tea plants grown in the same environment and were not infected with pathogens. Six trees were infested by tea green leafhoppers for 24 h and another 6 trees were used as control. The infested and control group were placed in two gauze cages separately (Figure 1). There are three replicates in in each group. An Ilumina NovaSeq platform was used to sequence the transcriptome of tea tree infested or not by tea green leafhoppers. A DP441 QIAGEN RNAprep Pure Plant Plus Kit was used to extract all of the RNAs. NEBNext Ultra TM RNA Library Prep Kit for Illumina (New England Biolabs, United States) was used to generate sequecing libraries, and we purified the mRNA. We then fragmented it in NEBNext First Strand Synthesis Reaction Buffer. First strand and second strand cDNA were synthesized using random hexamer primers, M-MuLV Reverse Transcriptase, and DNA Polymerase I and RNase H, respectively. Finally, the library preparations and pair-end reads (PE150) were produced. Tandem mass tag (TMT) and liquid chromatography–tandem mass spectrometry (LC–MS/MS) were used to quantitatively analyze proteome. The tea tree leaves were frozen in liquid nitrogen and dissolved in lysis buffer. The quality of extracted proteins was detected by SDS-PAGE. We added 10 mM dithiothreitol to 100 μg of proteins, which was incubated for 1 h at 37°C and followed by alkylation with 40 mM iodoacetamide for 45 min in the dark at room temperature. Trypsin (150 enzyme to protein) was added to the samples, which were then incubated overnight at 37°C. The reaction was terminated by the addition of 0.1% formic acid (FA). Next, samples were desalted using a C_18_ chromatographic column, washed with 70% acetonitrile (ACN), and vacuum freeze-dried according to the manufacturer’s instructions for the TMT kit. The LC–MS/MS spectra were then determined using XBridge Peptide BEH 5um C_18_. The separated peptides were parsed using an Orbitrap Exploris^™^ 480 mass spectrometer. Ultra-performance liquid chromatography-quadrupole time-of-flight mass spectrometry (UPLC-QTOF-MS) was used to analyze the metabolome. The LC/MS system for metabolomics analysis was composed of a Waters Acquity I-Class PLUS ultra-high-performance liquid tandem Xevo G2-XS QT of high-resolution mass spectrometry. A Waters Xevo G2-XS QTOF high-resolution mass spectrometer can collect primary and secondary mass spectrometry data in the MSe mode under the control of the acquisition software (MassLynx V4.2, Waters, United States). Dual-channel data acquisition can be performed simultaneously on both low collision energy and high collision energy in each data acquisition cycle. The low collision energy is 2 V, the high collision energy range is 10 ~ 40 V, and the scanning frequency is 0.2 s to obtain a mass spectrum. The settings of the ESI ion source were as follows: capillary voltage: 2,000 V; cone voltage: 30 V; ion source temperature: 150°C; dissolvent gas temperature 500°C; backflush gas flow rate: 50 l/h; and desolventizing gas flow rate: 800 l/h.

### Genome sequencing and assembly

Nanopore sequencing of 2 μg of gDNA was prepared using an NEB Next FFPE DNA Repair Mix kit (New England Biolabs) and subsequently processed using the ONT Template prep kit (Oxford Nanopore Technologies). The large segment library was premixed with loading beads and then pipetted onto a previously used and washed R9 flow cell. The library was sequenced on the ONT PromethION platform with the Corresponding R9 cell and ONT sequencing reagents kit (EXP-FLP001.PRO.6; Oxford Nanopore Technologies). The genome was assembled based on three ways: initial wtdbg2 (Ruan and Li, 2020) assembly and then SMARTdenovo assembly (Liu et al., 2021), followed by error correction using racon (Vaser et al., 2017) software and adjustment by Pilon (Walker et al., 2014) software. The assembly results were evaluated by the ratio of sequencing reads, Core Eukaryotic Genes Mapping Approach (CEGMA; Parra et al., 2007), and Benchmarking Universal Single-Copy Orthologs (BUSCO; Simão et al., 2015).

### Hi-C sequencing

We constructed Hi-C fragment libraries that ranged from insert sizes of 300–700 bp as illustrated in Rao et al. (2014) and sequenced them using the Illumina Novaseq 6,000 System (San Diego, CA, United States). The low-quality reads were removed and the clean data truncated, and the trimmed reads were then aligned to the assembly genome using a Burrows-Wheeler Aligner (BWA; Li and Durbin, 2009). Only unique paired-end reads that could be aligned and had a mapping quality > 20 were conserved. The valid interaction pairs were employed to correct the scaffolds and then ordered by LACHESIS (Burton et al., 2013). Finally, the vast majority of the sequences were located on the chromosomes.

### Repeat annotation

We integrated *de novo* and homology-based methods to recognize repetitive sequences in the genome of DuyunMaojian tea plants. We used LTR_FINDER (Xu and Wang, 2007) to look for homology. *De novo* predictions were conducted using RepeatScout (Price et al., 2005), and then PASTEClassifier (Hoede et al., 2014) and RepeatMasker were used to predict the repetitive sequences.

### Gene prediction and functional annotation

We used *ab initio*, homology, and RNA-Seq methods to predict the protein-coding genes of the tea plant. We used Genscan (Burge and Karlin, 1997), Augustus (Stanke and Waack, 2003), GlimmerHMM (Majoros et al., 2004), GeneID, and SNAP (Korf, 2004) to perform *ab initio* predictions. We compared the protein-coding genes from *Arabidopsis thaliana*, black cottonwood (*Populus trichocarpa*), coffee (*Coffea canephora*), rice (*Oryza sativa*), and kiwifruit (*Actinidia chinensis*) to the DuyunMaojian tea plant genome. The homology methods were carried out using GeMoMa (Keilwagen et al., 2016, 2018) software. We used TransDecoder, GeneMarkS-T (Tang et al., 2015), and PASA (Campbell et al., 2006) to predict the transcriptome. We then integrated the three predictions described using EVM software. We annotated the tRNA genes using tRNAscan-SE (Lowe and Eddy, 1997). We recognized the microRNA and rRNA based on the Rfam (Griffiths-Jones et al., 2005) database. We predicted the pseudogenes using BLAST (Kent, 2002) and GeneWise. We functionally annotated the protein-coding genes of the tea plant genome by performing BLAST searches against the NR, gene ontology (GO), EuKaryotic Orthologous Groups (KOG), Kyoto Encyclopedia of Genes and Genomes (KEGG), and TrEMBL databases.

### Comparative genomic analysis

We applied Orthofinder v2.5.1 software to recognize gene families of the tea plant and 14 other plant species, including Shuchazao, Yunkang10, DASZ, *Camellia oleifera Abel*, kiwifruit (*Actinidia chinensis*), coffee (*C. canephora*), *Arabidopsis thaliana*, cacao (*Theobroma cacao*), blueberry (*Vaccinium corymbosum*), *Rhododendron delavayi*, rice (*Oryza sativa*), apple (*Malus domestica*), grape (*Vitis vinifera*), *Citrus clementina*. We generated high-quality single-copy genes that were used to build the phylogenetic tree among the 15 species described above. The expansion or contraction events of gene families were computationally identified by CAFE v4.2 (De Bie et al., 2006). The synonymous substitution rates (*Ks*) of genes were calculated using wgdv1.1.132. We searched for LTR-RT sequences using LTR_FINDERv1.07 and LTRharvestv1.5.9 software. Collinearity analyses were conducted using VGSC35 software.

### The DEGs, DEPs and differential metabolites of the transcriptomic, proteomic and metabolomics analyses

The level of expression of the transcriptome genes was calculated using fragments per kilobase of transcript per million mapped reads (FPKM). The DEGs were investigated through DESeq with fold change ≥ 2 and FDR < 0.01 defined as DEGs. TMT and liquid chromatography–tandem mass spectrometry (LC–MS/MS) were used to quantitatively analyze the proteins of tea leaves infested with tea green leafhoppers. Proteins with fold change ≥ 2 and FDR < 0.01 were assigned as DEPs. UPLC-QTOF-MS techniques were used to analyze the metabolites qualitatively and quantitatively. We used principal component analysis (PCA), orthogonal projections to latent structures-discrimination analysis (OPLS-DA), and fold change (FC) to filter out the differential metabolites. The protein–protein interaction network (PPIN) of the DEPs were constructed based on two method: (1) the Interolog (Matthews et al., 2001) method and BLAST were conducted between the DEPs and STRING databases (Szklarczyk et al., 2021); and (2) The existing DEP PPINs were extracted from STRING databases and TeaGPIN (Singh et al., 2021). We obtained the DEP PPINs by integrating the three DEPs PPINs.

## Results

### Chromosome-scale assembly and annotation of the DuyunMaojian tea tree genome

**Figure 2:**
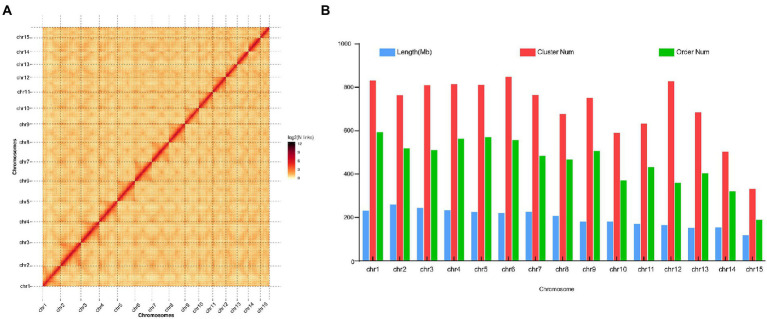
Hi-C heatmap based on the chromosome-scale assembly of the DuyunMaojian tea plant genome. **(A)** The heatmap represents the contact matrices generated by aligning the Hi-C data to the chromosome-scale assembly of the DuyunMaojian tea plant genome. **(B)** The assembly data statistics of each chromosome of the DuyunMaojian tea plant genome resulting from Hi-C.

The genome of the tea plant designated DuyunMaojian was sequenced and assembled using a combination of ONT and Hi-C. ONT produced 289.67 Gb of clean data, and the sequencing depth was 85.56 X (Supplementary Tables S1–S3). We got 3.35 Gb genome assembly and contig N50 was 778.61 kb. The GC content was 38.56% (Table 1). To validate the genome assembly quality, we first mapped all the high-throughput clean reads (916,216,962) to the assembled genome, the mapping rate was 91.66% and a proper mapping rate was 85.47% (Supplementary Table S4). Secondly, a CEGMAv2.5 result demonstrated that 90.61% of the core genes were found in the DuyunMaojian tea tree genome (Supplementary Table S5). Third, BUSCO v2.0 data demonstrated that 87.78% of the key genes were located in the assembly (Supplementary Table S6). A Hi-C analysis (Supplementary Tables S7–S10) was introduced to enhance the quality of tea plant genome and build a chromosome-scale assembly. After Hi-C assembly and manual adjustment, 2.97 Gb of the genome was mapped to 15 pseudo-chromosomes that anchored 96.35% of the assembled sequences and 2.79 Gb can confirm the order and direction (Figure 2). The redundant sequences from the heterozygous genome were removed by the Hi-C heat map, and the final genome was 3.08 Gb. After error correction, the contig N50, scaffold N50 and GC contents were 723.7 kb, 207.72 Mb and 38.54%, respectively (Table 1). The Hi-C heatmap perfectly showed 15 pseudo-chromosomes, which were designated chr1 to chr15, and the longest and shortest were chr2 and chr15 at 260,782,366 bp and 117,955,952 bp, respectively.

**Table 1:** Summary statistics of the DuyunMaojian tea plant assembly genome compared with those of other cultivars and species.

Genomic feature	DuyunMaojian using HIC	Shuchazao	Yunkang 10	DASZ	*Camellia oleifera*
Genome size (Gb)	2.97	2.94	3.02	3.11	2.89
Contig number	15,771	7,031	258,790	5,453	4,075
Contig N50 (kb)	723.697	600.46	19.96	2589.771	1,002
Contig max (kb)	6,456.183	2,885.68	257.648	16,831.527	/
Scaffold N50 (Mb)	207.721	218.1	0.45	204.21	185.364
Scaffold max (Mb)	250.703	/	3.51	336.927	/
GC content (%)	38.54	38.25	39.62	38.98	37.51
Gene number	34,896	50,525	36,951	33,021	42,426
Average gene length (bp)	6,961.34	4,906	3,549	8,050	3,955
Average exon length (bp)	232.25	245	237	211.7	317
Average intron length (bp)	1,117.79	973	640	1330.34	776
Reference	This study	Xia et al. (2020a)	Xia et al., (2017)	Zhang et al. (2020a,b)	Lin et al. (2022)

We identified 34,896 protein-coding genes and 2.67 Gb (86.77%) of repetitive sequences in the genome. Of the 34,896 genes, 33,481(95.95%) were annotated with the GO, KEGG, KOG, Pfam, TrEMBL and NR databases and we identified 16,674 SSRs. The prediction of noncoding RNA genes produced 104 miRNA, 261 rRNA, and 669 tRNA (Supplementary Table S11). In addition, 6,502 pseudogenes were annotated in our genome. Finally, we obtained 2,997 motifs and 37,323 domains. Retrotransposons elements include LTR and non-LTR. The LTR-RT include *Copia* and *Gypsy*, while the non-LTR include LINE and SINE. *Copia* and *Gypsy* were the predominant LTR-RTs and accounted for 12.5 and 43.68% of the DuyunMaojian tea plant genome, respectively (Supplementary Table S12). LINE is an autonomously active transposable element in the tea plant genome and accounts for about 1.68% of the genome. There were 27,830 SINEs, which accounted for 0.17% of the DuyunMaojian tea plant genome. They are small retrotransposable elements, which do not encode any transposable genes that are active. *Copia*, *Gypsy*, LINE, and SINE contribute to the expansion of the DuyunMaojian tea plant genome. Thus, retrotransposon elements may perform important functions in the responses to external stimulus.

### Functional annotation of tea tree genome

**Figure 3:**
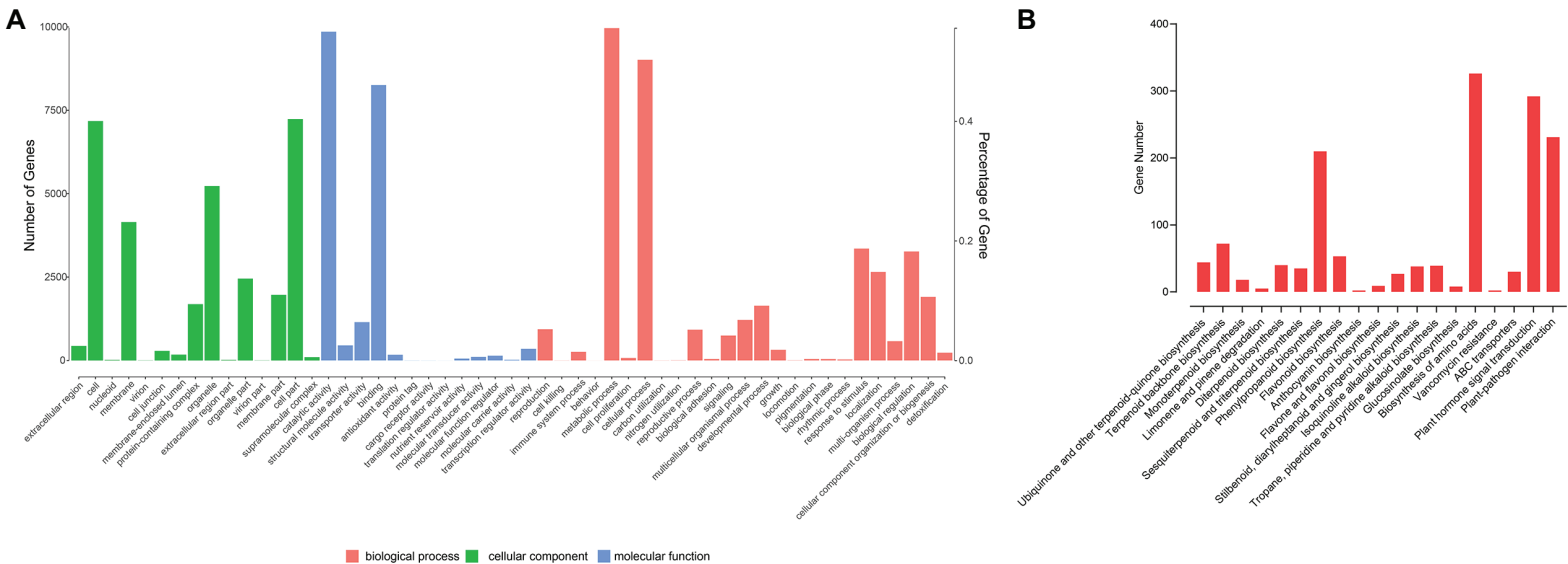
Gene annotation. **(A)** GO functional classification. **(B)** KEGG functional classification.

There were 17,919 genes annotated in the GO database (Supplementary Table S13). In cellular components, there were 15 secondary categories in which 7,177 genes, 7,236 genes and 5,229 genes were annotated to cell, cell part and organelle, respectively. In molecular function, 9,856, 8,257, and 1,149 genes were annotated to catalytic activity, binding, and transporter activity, respectively. In biological process, 9,963, 9,013, 3,354, 1,641, 318, 258, and 233 genes were annotated to metabolic process, cellular process, response to stimulus, developmental process, growth, immune system process, and detoxification, respectively (Figure 3A). A total of 11,407 genes were annotated to 128 pathways. A total of 44 genes were participated in ubiquinone and other terpenoid-quinone biosynthesis; 72 genes were took part in terpenoid backbone biosynthesis; 18 genes participated in monoterpenoid biosynthesis; 210 genes were took part in phenylpropanoid biosynthesis; 53 genes were involved with flavonoid biosynthesis; 70 genes were involved in α-linolenic acid metabolism; 292 genes participate in plant hormone signal transduction, and 231 genes participate in plant-pathogen interaction (Figure 3B). There were 17,700 genes annotated to the KOG database in all; 1,839 genes were classified to signal transduction mechanisms; 442 genes were classified into cell cycle, cell division, chromosome partitioning, and 213 genes were annotated to defense mechanism.

### Comparative genomic analysis

**Figure 4:**
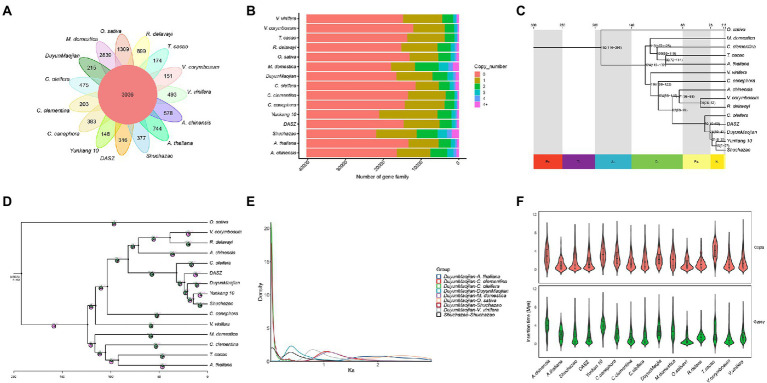
Comparative genomic analysis. **(A)**. A Venn diagram shows the shared and unique gene families among the DuyunMaojian and 14 other species. **(B)**. The copy number distribution of the DuyunMaojian tea plant and other 14 species. **(C)**. Phylogenetic tree of the DuyunMaojian tea plant and other 14 species. **(D)**. Expansion and contraction of gene families among the 15 plant species. **(E)**. Whole genome duplication events detected in the DuyunMaojian tea plant. **(F)**. Insertion times of copia and gypsy.

**Figure 5:**
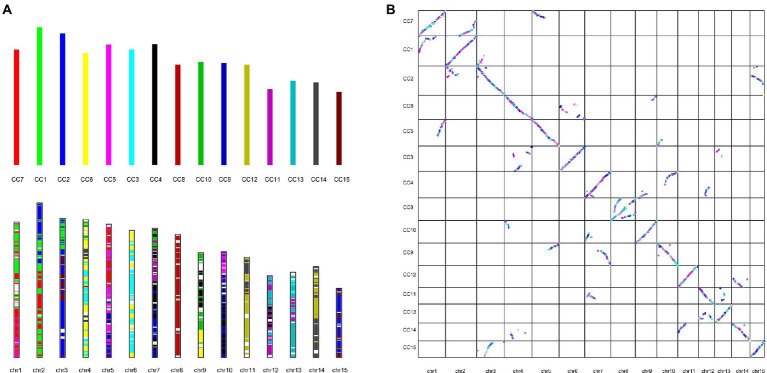
Collinearity analysis between DuyunMaojian and Shuchazao **(A)**. Evolutionary patterns of the chromosome-scale assembly of DuyunMaojian and Shuchazao. chr1-chr15 represent the 15 pseudo-chromosomes of DuyunMaojian. CC1-CC15 represent the 15 pseudo-chromosomes of Shuchazao. **(B)**. Comparison of chromosome-scale assembly of DuyunMaojian tea plant genome and Shuchazao.

Gene duplication within a species, evolution among species, and classification of species-specific genes (Figure 4A) were analyzed by comparing the genomes of DuyunMaojian tea plant with those of cacao (*T. cacao*), coffee (*C. canephora*), apple (*M. domestica*), *C*. *clementina*, kiwifruit (*A. chinensis*), blueberry (*V. corymbosum*), rice (*O. sativa*), *R. delavayi*, grape (*V. vinifera*), *A. thaliana*, Yunkang 10, Shuchazao, DASZ, and *C. oleifera*. The protein sequences of the DuyunMaojian tea plant and the other 14 species were classified using Orthofinderv2.5.1 (Emms, 2019). Thirty thousand and eight hundred and fifty three genes were grouped into 16,548 gene families (Table 2; Figure 4B). In addition, 215 gene families were specific to DuyunMaojian, and could be involved in specific biological processes, such as the large amount of synthesis of tea polyphenolics. IQ-TREEv1.6.11 (Nguyen and Schmidt, 2014), MAFFTv7.205 (Katoh et al., 2009), Gblocksv0.91b (Talavera and Castresana, 2007), and ModelFinder (Kalyaanamoorthy et al., 2017) software were used to study the evolutionary relationships between Duyun Maojian and the other 14 species using single-copy protein sequences. The results showed that DuyunMaojian had the closest relationship with already available tea tree genome of Shuchazao and Yunkang 10, followed by DASZ, *C. oleifera*, *V. corymbosum*, *R. delavayi*, *A. chinensis*, *C. canephora*, *V. vinifera*, *T. cacao*, *A. thaliana*, *C. clementina*, *M. domestica*, and *O. sativa* (Figure 4C). Shuchazao is evaluated to have diverged from Yunkang 10 about 15 (15–27) million years ago (MYA). DuyunMaojian is estimated to have diverged from Shuchazao and Yunkang 10 approximately 21 (8–37) MYA and split from DASZ approximately 24 (10–42) MYA. The cultivar DuyunMaojian is also evaluated to have diverged from *C. oleifera* approximately 32 (15–53) MYA and split from blueberry, *R. delavayi* and kiwifruit about 82 (69–97) MYA and from grape about 116 (108–122) MYA. We found that 201 gene families had expanded and 72 contracted in the DuyunMaojian tea plant genome using CAFÉv4.2 (De Bie et al., 2006; Figure 4D). The *Ks* of genes were calculated using wgdv1.1.1 (Zwaenepoel, 2019) and revealed that a recent whole-genome duplication (WGD) event (*Ks* = 0.39) was occurred in DuyunMaojian tea plant genome (Figure 4E). We searched for LTR-RT sequences using LTR_FINDERv1.07 (Xu and Wang, 2007) and LTRharvestv1.5.9 (Ellinghaus et al., 2008) software and the insertion time of *Copia* and *Gypsy* among the 15 species (Figure 4F). Collinearity analyses were conducted using VGSC (Xu et al., 2016) software, and there were 42,543 collinear genes between DuyunMaojian and Shuchazao (Figure 5). All these findings will greatly enhance our understanding of the diversification history of the DuyunMaojian tea plant genome.

**Table 2:** Classification of gene family.

Name	Total gene	Cluster gene number	Total family	Unifamily
*Vaccinium corymbosum*	20,771	18,538	12,109	151
*Rhododendron delavayi*	32,410	26,819	15,208	699
Shuchazao	50,525	48,637	21,900	377
*Theobroma cacao*	21,254	20,614	13,712	174
DuyunMaojian	34,896	30,853	16,548	215
*Oryza sativa*	28,317	24,773	13,093	1,309
*Arabidopsis thaliana*	27,533	24,544	13,398	744
*Malus domestica*	55,414	43,649	17,974	2,839
*Vitis vinifera*	29,421	23,785	14,808	493
*Coffea canephora*	25,100	22,141	14,306	383
*Citrus clementine*	22,207	20,342	13,495	203
*Actinidia chinensis*	39,444	30,001	16,420	578
Yunkang 10	36,951	31,246	21,130	148
DASZ	28,981	24,339	14,630	316
*Camellia oleifera*	33,173	31,856	11,504	475

### Multi-omics analysis of the response of tea plants to infestation with tea green leafhoppers

Tea green leafhopper (Liu et al., 2017; Yang, 2017) can significantly reduce the yield and quality of tea during the growth of plant. These insects can cause a loss of 11–55% in tea yields in the absence of effective means of prevention and treatment. Thus, we also used the tea plant transcriptome, proteome and metabolome in combination with its genome to analyze the DEGs, DEPs and differential metabolites after leafhopper infestation. We paid more attention on tea plants responds to infestation with tea green leafhoppers in a short time rather than a long-term mechanism. At the same time, we are trying to screen for the key DEGs, DEPs, and differential metabolites between the two groups. Therefore, we only selected 24 h for comparison of two groups after infestation by tea green leafhoppers. There were 1,575 DEGs, 871 DEPs, and 41 differential metabolites, respectively. A total of 281 DEGs, 353 DEPs, and eight differential metabolites identified through these processes were annotated to the KEGG database, respectively (Supplementary Tables S14–S16). We found that phenylpropanoid biosynthesis pathway, flavonoid biosynthesis pathway, and α-linolenic acid metabolism were simultaneously identified in the transcriptome, proteome, metabolome and genome. Therefore, we hypothesized that proteins and metabolites involved in the three pathways play important roles in the resistance to tea green leafhopper. The results indicated that phenylpropanoid biosynthesis, α-linolenic acid metabolism, and flavonoid biosynthesis contained 50 DEPs that were filtered out and found in the tea plant transcriptome, proteome, metabolome and genome (Supplementary Table S17). Thus, we hypothesized that the three pathways and 50 DEPs play important roles in response to tea green leafhoppers, which will provide a theoretical foundation to breed tea plants resistant to insects and ultimately result in increases in tea yield and quality.

### Protein–protein interaction network analysis of the response of tea plants to infestation with tea green leafhoppers

**Figure 6:**
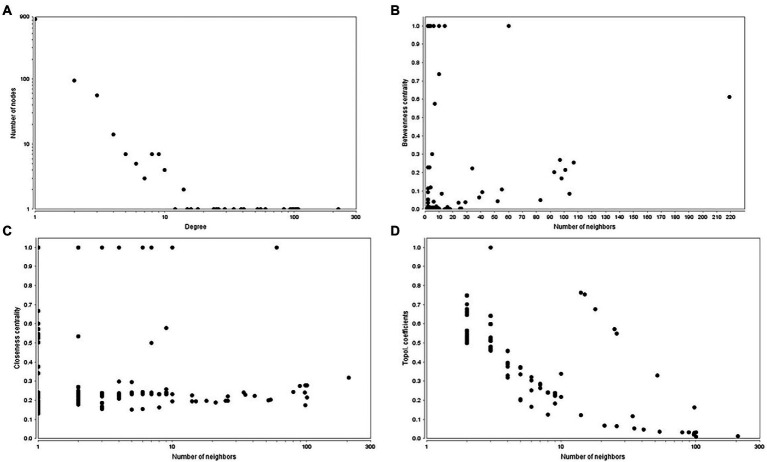
Topological properties of the PPINs. **(A)**. Degree of distribution. **(B)**. Betweenness centrality. **(C)**. Closeness centrality. **(D)**. Topological coefficient.

The predicted tea tree protein–protein interaction network (PPIN) was available in STRING database, TeaGPIN (Singh et al., 2021) and TeaLIPIN (Singh et al., 2019). There were 820 proteins and 1,067 protein–protein interactions (PPIs) in the predicted network based on the existing PPIN in the STRING database and the interolog method. We also extracted DEPs PPIN from TeaGPIN, in which there were 389 proteins and 583 PPIs (Supplementary Table S18). There were 362 shared PPIs in both network. We obtained the DEPs PPIN by integrating the two DEPs PPINs. In the merged DEPs PPIN (Supplementary Table S19), there were 1,047 nodes and 1,430 PPIs and a degree distribution of *P*(*k*) ≈ 53.574*x*^–1.008^, *R*^2^ = 0.684. The topological properties (Figure 6) of the PPIN showed that the degree of distribution of the proteins obeyed a power-law distribution (Figure 6A). There were a few proteins that have a high degree, which is a scale-free phenomenon. Highly connected proteins with central roles in the PPIN are hubs, and the degree of the hub proteins was calculated (Supplementary Table S20). These are the 21 proteins with the largest number of interactions, suggesting that they are also the most important proteins (hubs) in the PPIN. The betweenness centrality (Figure 6B) was a measure of a node’s centrality in a network equal to the number of shortest paths from all vertices to all the others that pass through that node. Its value was between 0 and 1. *C_b_*(*i*) = ∑*_m ≠ i ≠ n_*(*σ_mn_*(*i*)/*σ_mn_*), where *m* and *n* were the nodes in the network that differed from *i*, and *σ_mn_* denoted the number of the shortest paths from *m* to *n*. *σ_mn_*(*i*) was the number of shortest paths from *m* to *n* that *i* laid on. The closeness centrality (Figure 6C) of each node was also between 0 and 1 and was used to identify important positions within the network. *C_c_*(*i*) = 1/avg.(*S*[*u*,*v*]), where *S*[*u*,*v*] was the length of shortest path between the nodes *u* and *v*. *C_c_*(*i*) of node *i* is the reciprocal of the average shortest path length. The topological coefficient (Figure 6D) of protein *i* is *T_i_* = avg.(*J*(*i*,*j*))/*n_i_*, and *J*(*i*,*j*) that indicate that all nodes *j* share at least one neighbor with *i*. The topological coefficient decreases with the increasing number of neighbors, revealing that the nodes with many neighbors are not artificially clustered together. Thus, the merged DEPs PPINs of the 50 DEPs is reliably based on the topological property analysis described above. We hypothesized that the 21 hubs with the largest degrees, particularly peroxidase, played important roles in the response to tea green leafhopper infestation.

## Discussion

We reported the assembly of DuyunMaojian tea plant genome using a combination of the ONT PromethION^™^ combined with Hi-C technology, and the final genome was 3.08 Gb. A total of 2.97 Gb of the genome was mapped to 15 pseudo-chromosomes, among which 2.79 Gb can confirm the order and direction. The contig N50 was 723.7 kb, which was longer than that of Yunkang 10 (Xia et al., 2017) and shorter than that of Shuchazao (Xia et al., 2020a). The GC contents were 38.54%, which was consistent with those of the Yunkang 10 and Shuchazao genomes (Xia et al., 2020a). The ratio of *Copia* was higher than those in the CSSV1.1 genome, and the ratio of *Gypsy* was lower than those in the CSSV1.1 genome. In addition, we identified 16,674 SSRs, which was far less than those of the CSSV1.2 and CSA genomes.

A comparative genomics analysis showed that DuyunMaojian was the most closely related to Shuchazao and Yunkang 10, followed by DASZ, and *C. oleifera*. DuyunMaojian was evaluated to have diverged from *C. oleifera* approximately 32 (15–53) MYA, split from blueberry, *R. delavayi*, and kiwifruit about 82 (69–97) MYA and from grape about 116 (108–122) MYA. These estimates are similar to those of earlier studies (Shi et al., 2010; Fiz-Palacios et al., 2011; Wu, 2019). The *Ks* of genes were calculated using wgdv1.1.1 (Zwaenepoel, 2019) and revealed that a recent whole-genome duplication (WGD) event (*Ks* = 0.39) occurred in the DuyunMaojian tea plant genome, which was consistent with the findings of previous studies (Xia et al., 2017, 2020a; Wang et al., 2020). These data provide theoretical references for the cultivation of excellent tea varieties.

There were 33,481 annotated genes in the assembly genome, which were lower than those in the CSSV1.2 genome (Xia et al., 2020a) and higher than those in the CSSV1.1 genome (Wei et al., 2018). Tea plant leaves contain an extraordinarily high level of flavonoids that contribute to its health benefits and significantly affect its flavor, taste, and mouthfeel. Flavonoids, particularly flavonol glycosides and catechins, are the major source of the bitterness and astringency of tea. There were 53 genes annotated to flavonoid biosynthesis, 210 genes annotated to phenylpropanoid biosynthesis, and 70 genes annotated to α-linolenic acid metabolism in the tea plant genome, which contained 50 DEGs filtered out that were identified in the transcriptome, proteome and metabolome. Thus, we hypothesized that the three pathways (Zhao et al., 2020) and 50 DEGs played important roles in the response to tea green leafhopper infestation.

**Figure 7:**
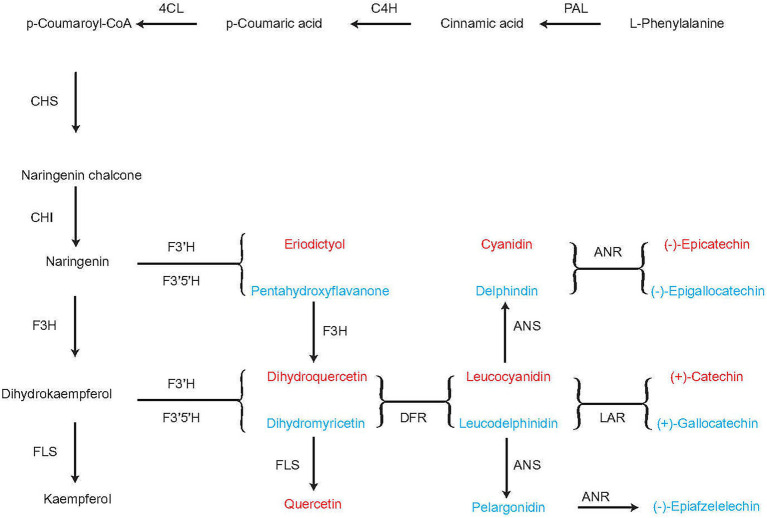
Flavonoid biosynthesis pathway in the tea plant. PAL, phenylalanine ammonia lyase; C4H: cinnamate 4-hydroxylase; 4CL: 4-coumarate-CoA ligase; CHS, chalcone synthase; CHI, chalcone isomerase; F3H, flavanone 3-hydroxylase; DFR, dihydroflavonol 4-reductase; ANR, anthocyanidin reductase; F3′5′H, flavonoid-3′5′-hydroxylase; F3’H, flavonoid 3′-hydroxylase; FLS, flavonol synthase; LAR, leucoanthocyanidin reductase.

When the tea plants were infested with tea green leafhoppers, they were influenced by DEPs, such as peroxidase. The putative flavonoid biosynthesis *via* the phenylpropanoid pathway are converted from cinnamoyl-CoA to pinobanksin 3-acetate by the enzymes chalcone synthase (CHS), chalcone isomerase (CHI), and flavanone-3-hydroxylase (F3H). L-Phenylalanine can be converted to *p*-coumaroyl-CoA through the subsequent action of phenylalanine ammonia lyase (PAL), cinnamate-4-hydroxylase (C4H), and 4-coumarate-CoA-ligase (4CL). *p*-Coumaroyl-CoA can be converted to naringenin through the action of CHS and CHI. Naringenin can be converted to either (−)-epicatechin through the subsequent actions of flavonoid 3′-hydroxylase (F3’H), flavonoid 3′, 5′-hydroxylase (F3′5′H), and anthocyanidin reductase (ANR) or to (−)-epigallocatechin through the subsequent action of 3′-hydroxylase (F3′H), flavonoid 3′, 5′-hydroxylase (F3′5′H), and anthocyanidin reductase (ANR). F3H catalyzes naringenin to dihydrokaempferol, which can be converted to either kaempferol through the action of flavonol synthase (FLS), or to dihydroquercetin, leucocyanidin, and finally (+)-catechin through the subsequent actions of F3′H, F3′5′H, dihydroflavonol 4-reductase (DFR), and leucoanthocyanidin reductase (LAR) or to (+)-gallocatechin through the subsequent actions of F3′H, F3′5′H, DFR and LAR. Naringenin can be converted either eriodictyol or pentahydroxyflavanone by the enzymes F3′H and F3′5′H, respectively. Eriodictyol and pentahydroxyflavanone can subsequently be converted to dihydroquercetin and dihydromyricetin by F3H, respectively, and finally converted to quercetin by FLS. DFR catalyzes the transformation of dihydromyricetin to leucodelphinidin, which can be converted to (−)-epiafzelechin by ANS and ANR (Figure 7). Thus, in the flavonoid biosynthesis pathway, we obtained (−)-epicatechin, (−)-epigallocatechin, (+)-catechin, (+)-gallocatechin, (−)-epiafzelelechin, kaempferol, and quercetin, which play important roles in improving the quality of tea and enabling it to respond to infestation by tea green leafhoppers. This result was consistent with those of previous studies (Treutter, 2006; Wang et al., 2016; Mei et al., 2017; Zhao et al., 2020).

**Figure 8:**
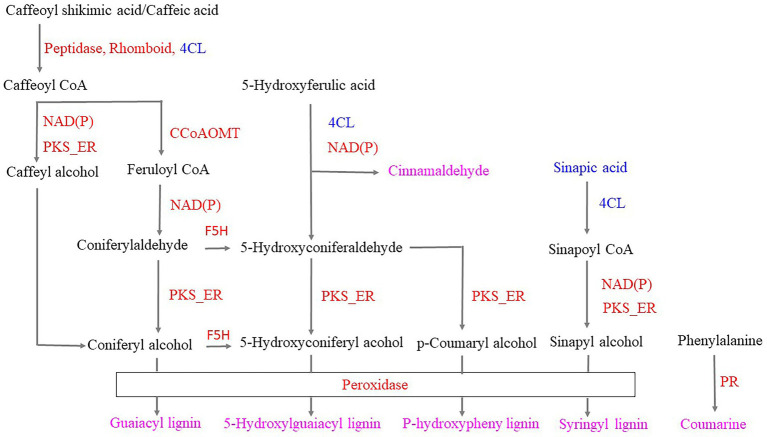
Responses of the phenylpropanoid biosynthetic pathway to infestation with tea green leafhoppers. Note: red proteins represent upregulation; blue proteins represent downregulation; pink metabolites were obtained in response to tea green leafhopper. PR: protein kinase; 4CL: 4-coumarate-CoA ligase; NAD(P): NAD(P)-bd_dom domain-containing protein; PKS_ER: PKS_ER domain-containing protein; Peptidase: peptidase_M16 domain-containing protein; Rhomboid: rhomboid domain-containing protein; CCoAOMT: caffeoyl-CoA o-methyltransferase.

Infestation of the tea plants with tea green leafhoppers resulted in the following changes in the phenylpropanoid biosynthesis pathway: (1) Phenylalanine can be converted to coumarin by upregulating protein kinase and other enzymes. (2) 5-Hydroxyferulic acid can be converted to cinnamaldehyde based on the downregulation of 4CL and the upregulation of NAD(P). (3) 5-Hydroxyferulic acid can be converted to *p*-coumaryl alcohol by the subsequent action of the downregulated 4CL, upregulated NAD(P), and upregulated PKS_ER. Peroxidase then catalyzes coumaryl alcohol to *p*-hydroxyphenyl lignin. (4) Caffeoylshikimic acid/caffeic acid can be converted to caffeoyl CoA through the action of upregulated peptidase, Rhomboid/downregulated 4CL. Caffeoyl CoA can be converted to either caffeoyl alcohol and coniferyl alcohol through the subsequent action of upregulated NAD(P) and PKS_ER or to feruloyl-CoA through the upregulated CCoAOMT and converted to coniferyl aldehyde through the action of upregulated NAD(P). Upregulated PKS_ER then catalyzes coniferyl aldehyde to coniferyl alcohol. Finally, coniferyl alcohol was converted to guaiacyl lignin through the action of upregulated peroxidase. (5) Coniferyl aldehyde and coniferyl alcohol can be converted to 5-hydroxyconiferaldehyde and 5-hydroxyconiferyl alcohol, respectively, through the action of upregulated F5H. Moreover, on the basis of downregulated 4CL, upregulated NAD(P) and PKS_ER, 5-hydroxyconiferaldehyde and 5-hydroxyconiferyl alcohol were obtained, which were catalyzed by the upregulated peroxidase, resulting in 5-hydroxyguaiacyl lignin. (6) Sinapic acid was downregulated and could be converted to sinapylCoA through the action of downregulated 4CL, and sinapylCoA can be converted to sinapyl alcohol by the subsequent catalysis of upregulated NAD(P) and PKS_ER. Finally, syringyl lignin was obtained by the catalysis of upregulated peroxidase. Thus, we hypothesized that when the tea plant was infested, peroxidase, coumarin, cinnamaldehyde, *p*-hydroxyphenyl lignin, guaiacyl lignin, 5-hydroxyguaiacyl lignin, syringyl lignin, and the phenylpropanoid biosynthetic pathway were used to respond to the tea green leafhopper (Figure 8), which was in accordance with the previous studies (Zhou et al., 2019; Natukunda, 2021; Souza et al., 2021; Zhang et al., 2022). These findings suggest that the resistance of tea plants to tea leafhopper could be obtained by inducing the synthesis of lignin, which could have potential applications in preventing insect infestations on tea.

**Figure 9:**
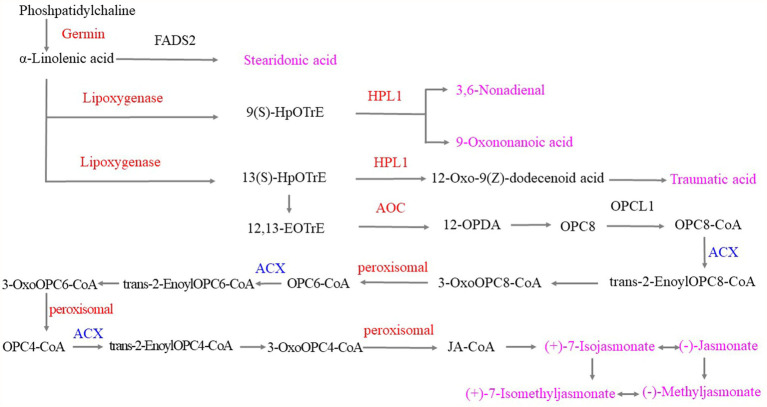
α-Linolenic acid metabolism in response to tea green leafhopper infestation in tea plants. Note: the red genes represent upregulation; the blue genes represent downregulation, and the pink metabolites were obtained in response to tea green leafhopper infestation.

The α-linolenic acid metabolic pathway played important roles when the tea plants were infested with tea green leafhoppers. Under the catalysis of upregulated germin-like protein, phosphatidylcholine was converted to α-linolenic acid and then converted to stearidonic acid, which was used to respond to tea green leafhoppers. Moreover, α-linolenic acid can be either converted to 9(S)-hydroperoxyoctadecatrienoic acid (HpOTrE) by the upregulation of lipoxygenase or to 3,6-nonadienal and 9-oxononanoid acid by the upregulation of hydroperoxide lyase 1 (HPL1). Next, α-linolenic acid was catalyzed by lipoxygenase, HPL1 and other enzymes to produce traumatic acid. Moreover, responses to the infestation of tea green leafhoppers included the catalysis of α-linolenic acid by upregulated lipoxygenase to obtain 13(S)HpOTrE, which was catalyzed by the related enzyme and then catalyzed the production of 12,13-EOTrE. Upregulated allene-oxide cyclase (AOC) catalyzed the transformation of 12,13-EOTrE to 12-OPDA, and 12-OPDA was then converted to (+)-7-isojasmonate, (−)-jasmonate, (+)-7-isomethyljasmonate, and (−)-methyl jasmonate by the downregulated ACX and upregulated peroxisomal, which can interconvert these compounds. Thus, we hypothesized that stearidonic acid, 9(S)-HOTrE, 3,6-nonadienal, 9-oxononanoid acid, 10-OPDA, traumatic acid, (+)-7-isojasmonate, (−)-jasmonate, (+)-7-isomethyl jasmonate, and (−)-methyl jasmonate played important roles in the response to tea green leafhopper infestation, which was consistent with the findings of previous studies (Nafie and Hathout, 2011; Xin et al., 2016; Zhao et al., 2020; Figure 9).

In the PPIN, we hypothesized that the hubs DEPs (Supplementary Table S20) and peroxidase in particular, may play important roles in the response to infestation by tea green leafhoppers.

In brief, the chromosome-scale genome of tea plant is highly promising to help understand the evolution of tea genome and then discover how the tea plant responds to tea green leafhopper. Our results suggest that (1) Duyun Maojian had the closest relationship with Shuchazao and Yunkang 10, followed by DASZ, and *C. oleifera*. (2) A recent whole-genome duplication (WGD) event (*Ks* = 0.39) occurred in the DuyunMaojian tea plant genome (3) The tea plant could become resistant to tea leafhoppers by the biosynthesis of lignin, which could have potential application values to prevent insect infestations on tea plants. (4) (−)-Epicatechin, (−)-epigallocatechin, (+)-catechin, (+)-gallocatechin, (−)-epiafzelelechin, kaempferol, and quercetin played important roles in the response to tea green leafhopper infestation. (5) Stearidonic acid, 9(S)-HOTrE, 3,6-nonadienal, 9-oxononanoid acid, 10-OPDA, traumatic acid, (+)-7-isojasmonate, (−)-jasmonate, (+)-7-isomethyljasmonate, and (−)-methyl jasmonate played important roles in response to tea green leafhopper infestation. (6) The hubs DEPs and peroxidase, in particular, may play important roles in the response to the tea green leafhopper infestation.

## Data availability statement

The datasets presented in this study can be found in the NCBI Sequence Read Archive under the BioProject accession number PRJNA841059. The DuyunMaojian tea plant gene and functional annotations are available in the figshare database (DOI: doi.org/10.6084/m9.figshare.19972808.v1).

## Author contributions

LL designed the study. DW and RL contributed to the sample preparation and genome sequencing. FW, BZ, and ML conducted the genome assembly, performed genome annotation and interpreted comparative genomic analysis. FW, BZ, XY, HP, RM, and ZC performed multi-omics analysis. FW wrote the manuscript. LL, BS, and FW modified the manuscript. All authors contributed to the article and approved the submitted version.

## Funding

This work was supported by the National Natural Science Foundation of China (grant number 32260080 and 31900486); Guizhou Provincial Science and Technology Foundation [(2019)1298]; Guizhou Provincial Education Department [ZDXK(2016)23, (2016)020, QNYSKYPT2018007, (2020)071, and (2015)68)]; the Scientific Research Project of Qiannan Normal University for Nationalities (QNYSKYTD2018011, Qnsyk201605, QNSY2018BS018, 2018xjg0520, QNYSXXK2018005, QNSY2018ZJ006, and 2019xjg0303).

## Conflict of interest

ML was employed by Biomarker Technologies Corporation.

The remaining authors declare that the research was conducted in the absence of any commercial or financial relationships that could be construed as a potential conflict of interest.

## Publisher’s note

All claims expressed in this article are solely those of the authors and do not necessarily represent those of their affiliated organizations, or those of the publisher, the editors and the reviewers. Any product that may be evaluated in this article, or claim that may be made by its manufacturer, is not guaranteed or endorsed by the publisher.
