# Supplementary material for: Chromosome-scale genome assembly of Camellia sinensis combined with multi-omics provides insights into its responses to infestation with green leafhoppers
Source: Front Plant Sci. 2022 Sep 23;13:1004387. doi: 10.3389/fpls.2022.1004387 (PMC9539759; doi:10.3389/fpls.2022.1004387)
Supplement: Supplementary file 6 [file Data_Sheet_1.pdf]

Supplementary Material

Supplementary Table 1. Statistic of sequencing data of tea plant genome using ONT PromethION™

DataType	SeqNum	SumBase	N50Len	N90Len	Mean Len	MaxLen	MeanQual
Clean data	13 610 732	289 671 640 748	28 050	15 295	21 282	723 888	7.95

Supplementary Table 2. Statistic of read length distribution

Length	ReadsNum	TotalLength	Percent	AveLength
2000-5000	1 169 364	3 956 336 035	1.37%	3 382.32
5000-10000	1 220 095	8 945 491 981	3.1%	7 331.79
10000-20000	2 863 699	45 179 861 668	15.66%	15 776.74
20000-30000	4 191 515	103 209 499 279	35.78%	24 623.43
30000-40000	1 709 847	58 457 906 753	20.26%	34 188.96
40000-50000	756 974	33 644 120 326	11.66%	44 445.54
50000-60000	374 423	20 344 589 753	7.05%	54 335.84
60000-70000	148 208	9 505 783 940	3.29%	64 138.12
70000-80000	47 767	3 533 328 709	1.22%	73 970.07
>=80000	18 611	1 673 395 252	0.58%	89 914.31

Supplementary Table 3. Summary of contig level assembly of tea plant genome

Contig number	Contig length(bp)	Contig N50	Contig N90(bp)	Contig max(bp)	GC content(%)	Gap total length(bp)
16 593	3 346 579 149	778 605	88 481	7 715 254	38.56%	0

Supplementary Table 4. The rate of clean reads mapped to tea plant genome

Total reads	Mapped reads	Mapped(%)	Properly mapped reads	Properly mapped(%)
916 216 962	839 786 900	91.66%	783 084 078	85.47%

Supplementary Table 5. CEGMA evaluation of the genomic completeness of tea plant

	number	Proportion(%)
Complete CEGMA	415	90.61
Highly conserved CEGMA	187	75.40
Total CEGAM	458	100

Supplementary Table 6. BUSCO evaluation of the genomic completeness of tea plant

	Number	Proportion(%)

Complete BUSCO(C)	1264	87.78
Complete and single-copy BUSCO(S)	1164	80.83
Complete and duplicated BUSCO(D)	100	6.94
Fragmented BUSCO(F)	39	2.71
Missing BUSCO(M)	137	9.51
Total BUSCO groups	1440	100

Supplementary Table 7. Statistics of clean data from Hi-C mapped to genome

Mapping type	number	Ratio (%)
Total read pairs	1 419 689 361	100
Mapped reads	2 313 815 368	81.49
Unique mapped read pairs	446 076 186	31.42

Supplementary Table 8. Valid and invalid read pairs

type	number	Ratio (%)
Unique paired alignments	446 076 186	100
Valid interaction pairs	324 600 582	72.77
Dangling end pairs	60 331 419	13.52
Re-ligation pairs	5701 115	1.28
Self-cycle pairs	11 628 256	2.61
Dumped pairs	43 814 814	9.82

Supplementary Table 9. Hi-C cluster and order sequences of each chromosome in tea plant genome

group	Cluster number	Cluster length(bp)	Order number	Order length(bp)
Chr1	831	230 776 669	593	219 540 409
Chr2	763	260 782 366	519	250 651 633
Chr3	810	244 100 597	510	224 927 390
Chr4	815	233 667 602	567	222 906 260
Chr5	811	225 229 618	570	216 144 798
Chr6	849	220 888 383	557	207 665 262
Chr7	765	226 035 287	484	209 183 189
Chr8	677	207 306 623	467	198 804 852
Chr9	751	180 052 669	507	169 544 396
Chr10	590	180 202 317	371	171 047 584
Chr11	632	170 151 961	432	162 188 652
Chr12	828	165 731 616	360	137 989 047
Chr13	685	151 995 148	403	137 927 593
Chr14	503	154 351 489	321	147 318 784
Chr15	331	117 955 952	189	112 171 158
Total (ratio%)	10641(67.47)	2969228297(96.35)	6850(64.37)	2788011007(93.9)

Supplementary Table 10. Scaffold and contig level of Hi-C assembly in tea plant genome

Scaffold number	Scaffold length(bp)	Scaffold N50(bp)	Scaffold N90(bp)	Scaffold max(bp)	Gap total length(bp)
8 936	3 082 241 746	207 720 862	112 189 958	250 703 433	683 500
Contig number	Contig length(bp)	Contig N50(bp)	Contig N90(bp)	Contig max(bp)	GC content(%)
15 771	3 081 558 246	723 697	89 025	6 456 183	38.54

Supplementary Table 11. The prediction of non-coding RNA

RNA classification	Number	Family
miRNA	104	23
rRNA	261	4
tRNA	669	25

Supplementary Table 12. Summary of repeat annotation of tea plant

Type	number	length	Rate (%)
ClassI	3 845 995	2 399 926 399	77.86%
ClassI/DIRS	174 008	161 819 434	5.25
ClassI/LINE	154 603	51 858 159	1.68
ClassI/LTR	29 202	15 731 243	0.51
ClasssI/LTR/Copia	632 994	385 245 376	12.5
ClassI/LTR/Gypsy	1 157 301	1 346 291 092	43.68
ClassI/PLE/LARD	1 656 614	811 508 137	26.33
ClassI/SINE	27 830	5 234 238	0.17
ClassI/SINE/TRIM	81	30 643	0
ClassI/TRIM	11 983	9 110 800	0.3
ClassI/Unknown	1 379	331 277	0.01
ClassII	236 390	118 488 325	3.84
ClassII/Crypton	18	1 002	0
ClassII/Helitron	38 870	12 786 129	0.41
ClassII/MITE	7 366	2 667 763	0.09
ClassII/Maverick	1 801	4 590 389	0.15
ClassII/TIR	175 169	94 864 378	3.08
ClassII/Unknown	13 166	4 419 994	0.14
Potential host gene	39 203	11 243 532	0.36
SSR	16 674	17 076 311	0.55
Unknown	1 006 689	382 850 268	12.42
Total	5 144 951	2 674 585 076	86.77

Supplementary Table 13. Functional annotation of tea plant genes

Annotation database	Annotated number	Percentage (%)

GO annotation	17 919	55.59
KEGG annotation	11 407	35.39
KOG annotation	17 700	54.91
Pfam annotation	25 939	80.48
TrEMBL annotation	30 959	96.05
NR annotation	31 022	96.25
All annotation	31 194	96.78

Supplementary Table 17. 50 DEPs exist in phenylpropanoid biosynthesis, alpha-Linolenic acid metabolism, and flavonoid biosynthesis

Uniprot ACC	Uniprot ID	Protein name
A0A4S4D455	TEA001905	Uncharacterized protein
A0A4S4DD17	TEA003727	LRRNT_2 domain-containing protein
A0A4S4E4C5	TEA010681	Uncharacterized protein
A0A4S4EJ14	TEA011765	Uncharacterized protein
A0A4S4EA04	TEA012289	HSF_DOMAIN domain-containing protein
A0A4S4DFA1	TEA015906	Cyclin N-terminal domain-containing protein
A0A4S4CWR2	TEA020350	PPM-type phosphatase domain-containing protein
A0A4V3WP28	TEA027322	Mur_ligase_M domain-containing protein
A0A4S4CXN5	TEA029800	Aa_trans domain-containing protein
A0A4S4E0L4	TEA013436	Peroxidase
A0A4S4E216	TEA008699	Chlorophyll a-b binding protein
A0A4S4EA26	TEA011776	Uncharacterized protein
A0A4S4EHC7	TEA022375	Germin-like protein
A0A4S4D4X9	TEA018193	Uncharacterized protein
A0A4S4DY67	TEA000939	Uncharacterized protein
A0A4S4ED09	TEA007838	Uncharacterized protein
A0A4S4EI95	TEA010521	Lipoxygenase
A0A4V3WNP8	TEA018011	Allene-oxide cyclase
A0A4V3WPJ7	TEA011393	Uncharacterized protein
A0A4S4EAC6	TEA011691	Peptidase_M16 domain-containing protein
A0A4S4D4N7	TEA013065	Uncharacterized protein
A0A4S4DT35	TEA019411	Uncharacterized protein
A0A4S4E967	TEA026798	NAB domain-containing protein
A0A4S4E988	TEA026805	Uncharacterized protein
A0A4S4ESZ8	TEA027441	Uncharacterized protein
A0A4V3WJ30	TEA027829	PPIase cyclophilin-type domain-containing protein
A0A4S4F0Q3	TEA028634	Uncharacterized protein
A0A4S4EN55	TEA024566	Peroxidase
A0A4S4D0Z1	TEA002780	Rhomboid domain-containing protein
A0A4S4DNL6	TEA003751	Protein kinase domain-containing protein
A0A4S4DUQ4	TEA008473	GH18 domain-containing protein
A0A4S4DYU8	TEA012735	LIM zinc-binding domain-containing protein

A0A4S4DQ18	TEA012891	Uncharacterized protein
A0A4S4EYY8	TEA015018	F-box domain-containing protein
A0A4S4DQS9	TEA022042	Uncharacterized protein
A0A4S4DUL8	TEA022897	Glutathione transferase
A0A4S4ESE8	TEA028587	PhoLip_ATPase_C domain-containing protein
A0A4S4DI86	TEA028682	DUF676 domain-containing protein
A0A4S4D0R4	TEA029796	NAC domain-containing protein
A0A4S4D0Z6	TEA024390	Peroxidase
A0A4S4D6Z6	TEA013258	Peroxidase
A0A4S4DKA0	TEA011333	Peroxidase
A0A4S4EBX2	TEA009181	PKS_ER domain-containing protein
A0A4S4ECG9	TEA024818	4-coumarate--CoA ligas
A0A4S4EHQ5	TEA028919	NAD(P)-bd_dom domain-containing protein
A0A4V3WMM3	TEA029791	Aldedh domain-containing protein
A0A4V3WQ16	TEA000358	Caffeoyl-CoA O-methyltransferase
A0A4V3WQ95	TEA009019	Peroxidase
A0A4S4D9R0	TEA017085	Uncharacterized protein
A0A4V6RY27	TEA013101	S-acyltransferase

Supplementary Table 20. The degree of the hub proteins in the merged DEPs PPIN

Uniprot ACC	Uniprot ID	Protein name	Degree
A0A4S4EAC6	TEA011691	Peptidase_M16 domain-containing protein	219
A0A4V3WMM3	TEA029791	Aldedh domain-containing protein	107
A0A4S4EHQ5	TEA028919	NAD(P)-bd_dom domain-containing protein	104
A0A4V3WJ30	TEA027829	PPIase cyclophilin-type domain-containing protein	101
A0A4S4D9R0	TEA017085	Uncharacterized protein	98
A0A4V3WP28	TEA027322	Mur_ligase_M domain-containing protein	97
A0A4V3WQ16	TEA000358	Caffeoyl-CoA O-methyltransferase	93
A0A4S4ECG9	TEA024818	4-coumarate--CoA ligas	83
A0A4S4DFA1	TEA015906	Cyclin N-terminal domain-containing protein	60
A0A4S4E216	TEA008699	Chlorophyll a-b binding protein	55
A0A4S4EN55	TEA024566	Peroxidase	52
A0A4S4D0Z1	TEA002780	Rhomboid domain-containing protein	41
A0A4S4DY67	TEA000939	Uncharacterized protein	39
A0A4S4D4N7	TEA013065	Uncharacterized protein	34
A0A4S4ED09	TEA007838	Uncharacterized protein	29
A0A4S4D6Z6	TEA013258	Peroxidase	26
A0A4S4D0Z6	TEA024390	Peroxidase	25
A0A4S4EI95	TEA010521	Lipoxygenase	24
A0A4S4DKA0	TEA011333	Peroxidase	18
A0A4S4E0L4	TEA013436	Peroxidase	15
A0A4V3WQ95	TEA009019	Peroxidase	14
